# Accuracy of Potassium Measurement Using Blood Gas Analyzer

**DOI:** 10.7759/cureus.23653

**Published:** 2022-03-30

**Authors:** Hatim Mahmoud, Zied Jaffar, Yousef M Al Alawi, Fatimah Al Alsuhaimi, Mohammed A A Khoja, Muath A Al-Ahmadi, Abdullah M Alattas, Mohammed F Alhusayni, Mohammed E Mahroos, Muath A Alrehaili

**Affiliations:** 1 Diabetes and Endocrinology, Prince Mohammed Bin Abdulaziz Hospital, Al-Madinah, SAU; 2 Infectious Disease, Prince Mohammed Bin Abdulaziz Hospital, Al-Madinah, SAU; 3 Internal Medicine, Prince Mohammed Bin Abdulaziz Hospital, Al-Madinah, SAU; 4 Medicine, Prince Mohammed Bin Abdulaziz Hospital, Al-Madinah, SAU

**Keywords:** diabetic ketoacidosis (dka), blood gas analyzer, accuracy, venous blood gas, potassium

## Abstract

Introduction: Newer blood gas analyzers can measure both blood gases and electrolytes in both arterial and venous blood samples. They are small, compact, and mobile point of care test (POCT) devices. They can produce results in as short as five minutes. We aimed at assessing the accuracy of potassium (K) level measured by gas analyzer (index test) by comparing that to the regular laboratory machine (reference standard) in our hospital. Our goal is to use POCT result of potassium so we may start insulin infusion within five to 10 minutes of arrival of diabetic ketoacidosis (DKA) patients to the emergency room (ER). It takes an average of 30 minutes to get the result using the reference standard machine. Potassium level is needed urgently in cases of DKA before initiating insulin infusion. That is true also during cardiopulmonary resuscitation (CPR) and while replacing K in severe hypokalemia and during the management of hyperkalemia.

Methods: We looked into the potassium results from 265 patients who had venous blood gas (VBG) or arterial blood gas (ABG) samples and compared that to results of potassium in venous blood samples of these same patients done simultaneously or within two hours. All patients who had blood gas and venous blood drawn simultaneously or within two hours were eligible irrespective of gender, age, diagnosis, and location in the hospital. Data were collected between January 2019 and June 2019. We excluded all cases that were receiving IV fluids, diuretics, or potassium supplements. Samples examined were from all different areas of the hospital including emergency room (ER), intensive care unit (ICU), and general floors. All ages and all diagnoses were included.

Results: We used the Bland-Altman method to analyze our data. More than 95% of the data fell within ± 2 standard deviations (S) of the mean difference strongly suggestive of agreement between the index test and the standard reference of the laboratory methods. The bias was 0.19. Lin’s concordance correlation coefficient was 0.6584.

Conclusion: Findings of this study support the use of POCT blood gas analyzer for measuring potassium when the results are needed urgently. When measuring potassium, blood gas analyzers are as accurate as automated analyzers. They produce results in five minutes or so and can be relied upon when potassium level is needed urgently. They are cost-effective and may be available at the bedside.

## Introduction

Potassium level is tightly controlled by different physiological and homeostatic mechanisms. Hypokalemia and hyperkalemia are reversible causes of cardiac arrhythmias [1]. Potassium is a major intracellular cation. In diabetic ketoacidosis (DKA), the increased osmolality of the extracellular fluid (ECF) draws water out of the cells leading to elevation of the intracellular concentration of potassium which in turn increases its gradient to the outside. Proteolysis and glycogenolysis secondary to insulin deficiency cause efflux of potassium. Acidosis contributes only a minor role in potassium distribution. Potassium is then lost as a result of vomiting and keto-anion excretion (anion excretion in the urine requires excretion of cations, potassium, and sodium). Osmotic diuresis is another mechanism of potassium loss. Secondary hyperaldosteronism due to volume depletion also causes potassium loss in the urine. While the total body potassium is depleted, patients with DKA can present with low, normal, or high potassium level [2]. Infusion of insulin and correction of acidosis drives the potassium into the cell [3]. This can result in severe life-threatening hypokalemia if the patient's potassium is already low. The American Diabetes Association (ADA) guidelines recommend that potassium level must be measured and corrected before insulin infusion is started [4]. A delay in initiation of insulin in DKA can cause the patient's condition to progress rapidly from being mild to moderate or even severe one requiring ICU admission, and that might also increase the risk of complications and prolonged hospital stay.

Blood gas analyzers can produce potassium results in about five minutes. This, along with the location of the machine in the ER, can help initiate insulin infusion within a few minutes of DKA presentation avoiding unnecessary delay. As a matter of fact, patients with DKA might at times be discharged home within a few hours of presentation if treatment is started promptly.

In this study, we aimed at establishing the accuracy of point of care test (POCT) gas analyzers in measuring potassium with the objective of using that instead of the costly and time-consuming reference standard laboratory machine at times when the result is needed urgently. The standard machine produces results in about 30 minutes, a delay that is not acceptable in life-threatening emergencies like DKA and cardiac arrest. During cardiopulmonary resuscitation (CPR), potassium abnormalities are either missed or treated empirically based on patient history, examination, and previously available laboratory results. Using gas analyzers, we can obtain results while CPR is being conducted leading to an accurate diagnosis and appropriate management that might save the patient's life.

## Materials and methods

This was a retrospective study. It was done after receiving approval from the ethics committee of King Abdullah International Research Center (KAIMRC). This research did not receive any specific grant from funding agencies in the public, commercial, or not-for-profit sectors. All patients who had blood gas and venous blood drawn simultaneously or within two hours were eligible irrespective of gender, age, diagnosis, and location in the hospital. Data were collected between January 2019 and June 2019 in our tertiary hospital with 250 beds (Prince Mohammed Bin Abdulaziz Hospital {PMBAH}, Al-Madinah, KSA). Data were collected by a group of internal medicine residents. Blood gas samples were drawn and analyzed by trained respiratory therapists. The gas analyzers were operated, maintained, and standardized according to the manuals. Venous samples were run in the main laboratory by trained personnel. The laboratory results and patients’ profiles were obtained from our electronic medical record system named BestCare (ezCaretech, South Korea). The sample size was 610 patients. Exclusion criteria include the presence of hemolysis in lab reporting, receiving diuretics, administration of any type of IV fluids during sample collection, administration of potassium supplement on the day of sampling, and samples collection of more than two hours apart (we thought that more than two hours might cause physiological or pathological differences in level of the electrolytes).

## Results

In this study, we reviewed 610 cases that had results for both venous blood gas (VBG) or arterial blood gas (ABG) and serum electrolytes. Out of these cases, only 265 met the inclusion criteria. The mean age was 42 (30) years and males accounted for 60% of the total cases (Table 1).

**Table 1:** Age distribution of patients

Age groups (years)	Number of patients (%)
< 15	79 (29.8)
15-39	44 (16.6)
40-59	38 (14.3)
> 60	104 (39.3)

**Figure 1:**
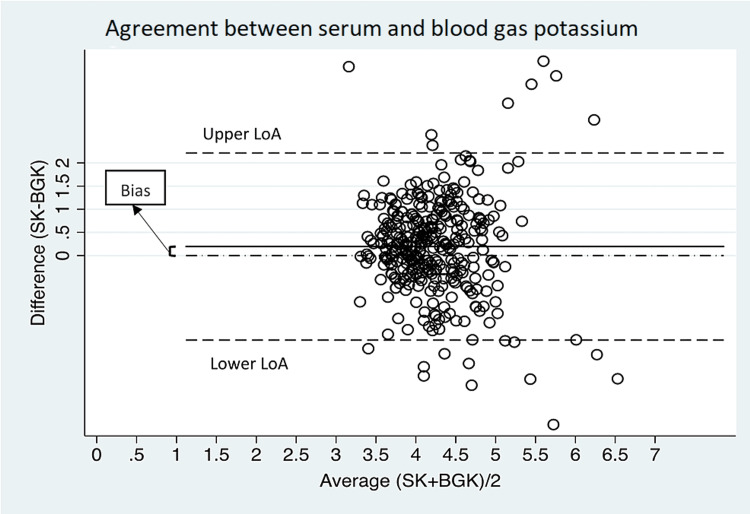
Plot of differences between standard reference and index test vs the mean of the two measurements The bias of 0.19 units is represented by the gap between the X-axis, corresponding to zero differences, and the parallel line to the X-axis at 0.19 units. SK: serum potassium; BGK: blood gas potassium; LoA: lower limit of agreement

The mean serum potassium (SK) and blood gas potassium (BGK) were 4.1 mmol/dl and 4.3 mmol/dl, respectively, while the median SK was 4.0 mmol/dl and that of BGK was 4.2 mmol/dl. When using a new method or instrument to measure a variable, we need to ensure that there is an agreement between the two methods, the new and the existing standard one. We used the Bland-Altman plot (B&A) method to describe or quantify the agreement between our BGK and SK levels; in other words, the agreement between the index test and the reference standard. Here, we plotted the difference of the two paired measurements against the mean of the two measurements, i.e., SK-BGK on the Y-axis against SK+BGK/2 on the X-axis. The resulting graph is a scatter plot XY (Figure 1).

In our study, the bias is represented by the gap between the X-axis, corresponding to zero differences, and the parallel line to the X-axis. B&A recommends that 95% of the data points should fall within ± 2 standard deviations (S) of the mean difference (Table 2) [5].

**Table 2:** Bland-Altman method - absolute values of bias and limits of agreement SK: serum potassium; BGK: blood gas potassium

Parameter	Estimate	Standard deviation	Standard error	95% CI
Bias (difference SK-BGK)	0.19	1.03	0.05	0.08 to 0.30
Lower limit of agreement	-1.82	-	-	-2.01 to -1.64
Upper limit of agreement	2.21	-	-	2.03 to 2.39

**Figure 2:**
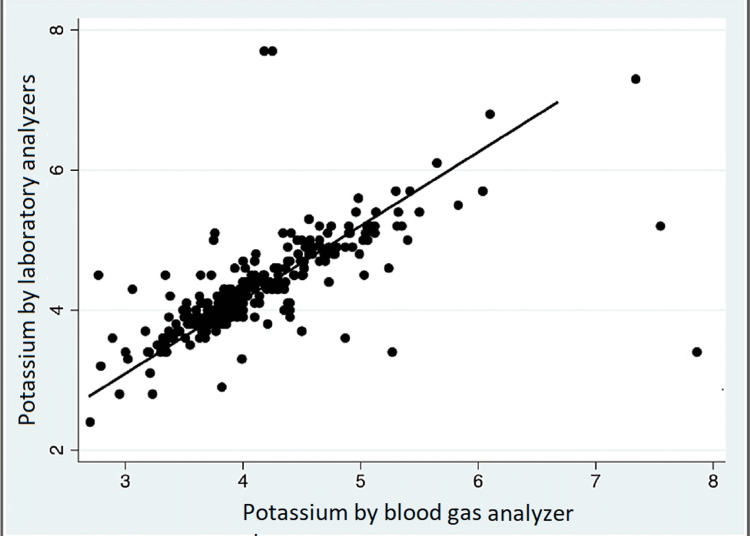
Relation between standard reference and index test measurements

We used computer software to calculate Lin’s concordance correlation coefficient (CCC) [6]. Like the correlation coefficient (r), CCC ranges from -1 to 1, with perfect agreement at 1. Our CCC for this study was 0.6584. Direct plotting of SK against BGK showed a linear regression correlation (Figure 2).

While this finding indicates the presence of a relationship between the two variables, it does not tell the difference between them. The correlation coefficient and regression technique can at times be misleading when assessing agreement, as they evaluate only the linear association of two sets of variables [5]. There were no adverse events from performing the index test or the reference standard.

## Discussion

The study results demonstrate strong agreement between the index test and the standard reference for potassium measurements as illustrated by the B&A plot (Figure 1). Although the linear regression graph is suggestive of obvious relationship or correlation between the two methods of measurement and supports our data, this statistical analysis is not appropriate for the evaluation of new instrument [7].

In our case, there is a clear agreement between our index and standard machines. The mean and the median of the measurements are not considered accurate or strong methods of comparison in such study, nevertheless, their obvious agreements add to the statistical significance of the results of B&A method in our study. Gas analyzers can give potassium results within five minutes while it usually takes up to 30 minutes or more using the main hospital laboratory system.

The result of our study adds to the existing literature that favors the use of gas analyzers in the measurement of potassium, reducing both cost and time. Luukkonen et al. found that the Epoc analyzer (SIEMENS, Germany) is accurate compared to Siemens Rapidlab 1265 (SIEMENS, Germany) and Rapidpoint RP500 (SIEMENS, Germany), and Siemens Dimension Vista (SIEMENS, Germany) and Sysmex XE-2100 analyzers (Sysmex Corporation, Japan), and they concluded that electrolytes measured by analyzers may be used for rapid measurement of the electrolytes in the ICU [8]. In addition, Walton et al. gathered 30 samples of blood and measured potassium and other electrolytes using ABL-70 point of care blood gas analyzer and found that the results were accurate, reliable, and consistent [9]. Using a rapid method to get the level of potassium will significantly impact the management of conditions where potassium results are needed urgently like in DKA before initiating insulin infusion. Also, during CPR, potassium results obtained by gas analyzer can have a leverage advantage on decision making. Prakash et al. analyzed 9398 matched pairs of blood gas analyzer and automatic analyzer machine in a central laboratory and they found high concordance of results of electrolytes in critically ill patients [10]. D'Orazio et al. found that blood gas analyzers can also be used to measure other electrolytes like calcium [11]. In another study by Jain et al., sodium and potassium were measured in 200 paired samples collected from ICU patients. Here potassium measurement, using ABG analyzer, was accurate when compared to the automated analyzer. Interestingly, sodium result was not as accurate [12]. 

One of the limitations of our study is that the results cannot be generalized because we used specific index machine (blood gas analyzer Cobas b 221; Roche Diagnostics, Switzerland) against specific standard machine (ARCHITECT c4000; Abbott, USA), and therefore every institute should run a similar study to test the agreement of the machines they use. Another limitation is that the paired samples in many patients were not taken at the same time as we accepted up to two hours in between. Also, assuming that the accuracy of the results is not affected by the underlying diagnosis is another limitation. Some physicians are still skeptical about using blood gas analyzers to measure electrolytes because there are only a few studies that support their accuracy. We think that our study adds to the existing evidence that these devices are accurate enough in measuring potassium. Hospitals can conduct their own studies to assess the accuracy of their devices and then introduce them to protocols for managing conditions like DKA and cardiac arrest.

## Conclusions

We found that there was no significant difference between the values of potassium measured by the blood gas analyzer and the automated analyzer. This study indicates that the gas analyzers may be reliable when potassium level is needed urgently. They produce results in five minutes or so. This can positively affect the management of conditions like DKA and cardiac arrest. The study adds to the available literature that encourages physicians to use POCT devices that are usually available at the bed side at an affordable cost. Hospitals need to run their own comparison studies to assure accuracy of their blood gas analyzers.
